# Prognostic value of glycolysis markers in head and neck squamous cell carcinoma: a meta-analysis

**DOI:** 10.18632/aging.202583

**Published:** 2021-02-26

**Authors:** Yanting Wang, Yuanyuan Li, Laibo Jiang, Xianyue Ren, Bin Cheng, Juan Xia

**Affiliations:** 1 Hospital of Stomatology, Sun Yat-Sen University, Guangzhou 510055, Guangdong, China; 2 Guangdong Provincial Key Laboratory of Stomatology, Guangzhou 510055, Guangdong, China; 3 Guanghua School of Stomatology, Sun Yat-Sen University, Guangzhou 510055, Guangdong, China

**Keywords:** glycolysis marker, prognosis, HNSCC, meta-analysis

## Abstract

Glycolysis markers including glucose transporter 1 (GLUT1), monocarboxylate transporter 4 (MCT4), hexokinase 2 (HK2), pyruvate kinase M2 (PKM2) and glucose transporter 4 (GLUT4) play vital roles in head and neck squamous cell carcinoma (HNSCC). However, their prognostic value in HNSCC is still controversial. In this meta-analysis, we searched the PubMed, Web of Science and Cochrane Library databases and included thirty-seven studies (3272 patients) that met the inclusion criteria. Higher expression levels of the glycolysis markers in tumor tissues correlated with poorer overall survival (OS; *P* < 0.001), disease-free survival (DFS; *P* = 0.03) and recurrence-free survival (RFS; *P* < 0.001) of HNSCC patients. Subgroup and sensitivity analyses demonstrated that higher expression levels of GLUT1 (*P* < 0.001), MCT4 (*P* = 0.002), HK2 (*P* = 0.002) and PKM2 (*P* < 0.001) correlated with poorer OS among HNSCC patients. Higher expression of MCT4 (*P* < 0.001) and PKM2 (*P* = 0.008) predicted poorer DFS among HNSCC patients. However, GLUT4 expression levels did not associate with clinical outcomes in HNSCC patients. These results demonstrate that glycolysis markers, such as GLUT1, MCT4, HK2 and PKM2, are potential prognostic predictors and therapeutic targets in HNSCC.

## INTRODUCTION

Head and neck cancer (HNC) is the sixth most common cancer worldwide, with nearly 600,000 new cases diagnosed annually [1, 2]. The most common type of HNC is squamous cell carcinoma (SCC), which arises from the stratified epithelium of oral cavity, nasopharynx, hypopharynx, oropharynx, or larynx [3]. The anatomy of the head and neck is complex and tumors arising from different sites of this region demonstrate unique histology, phenotype, tumorigenicity, and invasive properties [4]. Despite rapid advances in surgery and adjuvant therapy, the 5-year overall survival rate of HNC patients is approximately 50% [5]. Higher rates of local recurrence, secondary tumors, and distant metastasis contribute to increased mortality of HNC patients [6].

In normal healthy cells, glucose is oxidized completely to CO_2_ through the mitochondrial respiratory chain in the presence of oxygen to generate significant amounts of ATP or converted to lactic acid via glycolysis in oxygen-deficient conditions [7]. In the early 1900s, Otto Warburg discovered that cancer cells resort to the use of glycolysis as a way to generate ATP even when the oxygen is sufficient [8]. Aerobic glycolysis is extensively involved in the development and progression of most cancers, including head and neck squamous cell carcinoma (HNSCC).

Glucose transporter 1 (GLUT1), monocarboxylate transporter 4 (MCT4), hexokinase 2 (HK2), pyruvate kinase M2 (PKM2) and glucose transporter 4 (GLUT4) are key enzymes that regulate the rate of glycolysis [9–11]. GLUTs are mainly responsible for glucose uptake into cells [9]. MCT4 is typically involved in exporting excessive lactate out of the cells [10]. Hexokinases (HKs) catalyze the first step of glycolysis, which involves phosphorylation of glucose into glucose-6-phosphate [11]. Pyruvate kinase catalyzes the last step of glycolysis, during which the high-energy phosphate group is transferred to form Pyruvate and produce ATP [7]. These glycolytic enzymes play vital roles in several human cancers, and regulate proliferation, metastasis, and chemoresistance of cancer cells [9, 11–13]. Several studies have evaluated the prognostic roles of GLUT1, MCT4, HK2, PKM2 and GLUT4 in HNSCC, but the results are conflicting [14–41].

In this study, we performed systematic meta-analysis to evaluate the prognostic significance of glycolysis markers, namely, GLUT1, MCT4, HK2, PKM2, and GLUT4 in HNSCC.

## RESULTS

### Study characteristics

**Figure 1:**
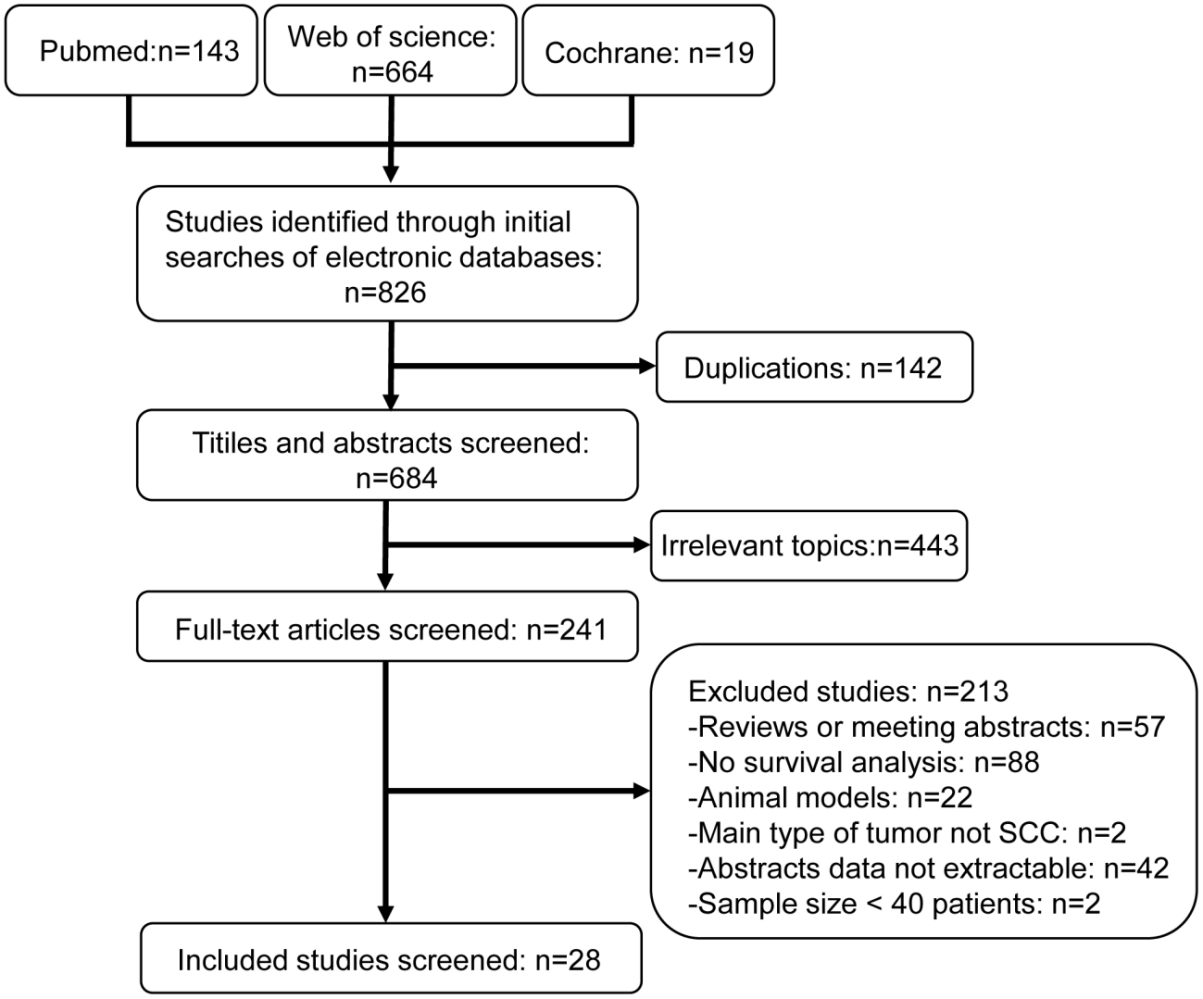
**Flow diagram shows selection strategy of studies included in this meta-analysis.**

The literature selection process is shown in Figure 1. The characteristics of included studies are listed in Table 1. We included 28 research articles published between the years 2000 and 2020 for the meta-analysis. These included 37 studies and 3272 patients. The quality of the included studies was assessed using Newcastle-Ottawa Scale (NOS) (Supplementary Table 1). Nineteen studies were conducted in Asia, whereas, the remaining studies were conducted in North and South America (n=7) and Europe (n=11). Majority of the studies assessed GLUT1 (n=19), whereas, the remaining studies assessed GLUT4 (n=4), MCT4 (n=4), HK2 (n=6), and PKM2 (n=4). The sample size of these studies varied from 33 to 274 HNSCC patients. Based on the median sample size, 18 studies were defined as large sample size studies (n>71) and the remaining 19 studies were defined as small size studies (n≤71). The prognostic value of specific glycolysis markers was investigated by evaluating overall survival (OS) in 21 studies, disease-free survival (DFS) in 9 studies, recurrence-free survival (RFS) in 3 studies, disease-specific survival (DSS) in 2 studies, and distant metastasis- free survival (DMFS) in 2 studies.

**Table 1:** Characteristics of included studies.

**Author year**	**Glycolysis marker**	**Country**	**Ethnicity**	**Tumor location**	**Follow-up (months)**	**Sample size**	**Gender (M/F)**	**Detection method**	**TNM stage**	**Cut-off value**	**Outcome**	**HR [95% CI]**	**Study design**	**NOS score**
Ayala 2010[14]	GLUT1	Brazil	Caucasian	OSCC	64.9(0.03-157.6)	142	112/30	TMA IHC	I-IV	≥10%	OS	2.07[1.23-3.46]	P	7
Baschnagel 2015[35]	GLUT1 HK2	America	Caucasian	HNSCC	35(1-93)	97	NA	IHC	I-IV	GLUT1:Score=3(0-3) HK2:Score≥1(0-3)	DFS	2.13[0.86-5.28] 1.01[0.50-2.05]	P	7
Brockton 2011[15]	GLUT1	Canada	Caucasian	HNSCC	NA	47	37/10	TMA IHC	II-IV	NA	OS	1.21[0.22-6.65]	P	6
Chang 2017[16]	GLUT4	China	Asian	HNSCC	190	90	81/9	IHC	I-IV	Score≥2(0-3)	OS RFS	3.31[1.28-8.55] 3.76[1.76-8.03]	P	8
Choi 2007[31]	GLUT1	Korea	Asian	OSCC	4.10-117.13	60	40/20	IHC	I-IV	≥60%	OS DFS	1.70[0.63-4.62] 1.62[0.78-3.34]	P	7
Curry 2013[36]	MCT4	America	Caucasian	OSCC	45(2.8-94.9)	42	27/15	IHC	I-IV	≥25%	DFS	10.36[2.56-41.94]	P	7
Deron 2011[38]	GLUT4	Belgium	Caucasian	TTSCC	49(1-123)	71	62/9	IHC	I-IV	Score≥2(0-15)	OS DFS	1.08[0.41-2.89] 0.78[0.32-1.92]	P	7
Eckert 2008[32]	GLUT1	Germany	Caucasian	OSCC	60	42	33/9	IHC	I-IV	Score≥6(0-12)	OS	5.05[2.05-12.45]	P	7
Eckert 2011[40]	GLUT1	Germany	Caucasian	OSCC	44.3	82	60/22	IHC	I-IV	Score≥9(0-12)	DSS	1.76[0.78-3.93]	P	7
Grimm 2014[37]	GLUT1	Germany	Caucasian	OSCC	52.26(46.21-58.31)	161	122/39	IHC	I-IV	Score≥1(0-9)	DFS	0.29[0.04-2.32]	P	8
Han 2012[17]	GLUT1	Korea	Asian	OSCC	40(9-113)	33	20/13	IHC	II	>10%	DFS OS	1.16[0.14-9.61] 12.46[0.67-231.55]	P	5
Jonathan 2006[41]	GLUT1	Netherlands	Caucasian	HNSCC	61.2	58	43/15	IHC	I-IV	Score≥2(0-3)	DMFS	4.67[0.33-65.13]	P	8
Krupar 2017[19]	GLUT1	Germany	Caucasian	HNSCC	60	73	67/7	IHC	II-IV	NA	OS	1.75[1.01-3.04]	P	6
Kunkel 2003[33]	GLUT1	Germany	Caucasian	OSCC	74(1-172)	118	88/30	IHC	I-IV	≥50%	OS	2.65[1.24-5.65]	P	7
Kunkel 2007[34]	GLUT1	Germany	Caucasian	OSCC	62(25-106)	40	33/7	IHC	I-IV	NA	OS	5.12[1.12-23.40]	P	6
Oliver 2004[39]	GLUT1	UK	Caucasian	OSCC	60-72	54	36/18	IHC	NA	Score≥2(0-3)	RFS	2.66[0.56-12.78]	P	6
Swartz 2016[18]	GLUT1	Netherlands	Caucasian	Oropharyngeal SCC	35(15.8-67)	274	190/84	TMA IHC	I-IV	>6%	OS	1.50[1.05-2.15]	P	7
Sweeny 2012[20]	MCT4	America	Caucasian	cSCC of the head and neck	NA	50	43/7	IF	III-IV	>50%	DSS	2.42[0.48-12.33]	P	7
Wang 2015[21]	PKM2	China	Asian	OSCC	51.4(3-78)	111	60/61	IHC	I-IV	Score≥4(0-12)	OS DFS	3.12[1.66-5.85] 2.53[1.01-6.37]	P	7
Wang 2017-1[22]	HK2	China	Asian	OSCC	71.3	137	89/48	IHC	I-IV	Score>4(0-9)	OS	2.15[1.07-4.31]	P	7
Wang 2017-2[23]	PKM2	China	Asian	OSCC	67	137	89/48	IHC	I-IV	Score>4(0-9)	OS	2.15[1.02-4.52]	P	7
Wu 2013[24]	GLUT1	China	Asian	LSCC	42.6(13-181)	49	43/6	IHC	I-IV	>10%	OS	3.46[0.73-16.25]	P	7
Xiao 2014[25]	HK2	America	Caucasian	NPC	69.72	41	38/9	IHC	I-III	NA	OS	2.05[1.01-4.16]	P	4
Yuan 2014[26]	PKM2	China	Asian	OSCC	46.8(2-80)	63	37/26	IHC	I-IV	Score≥4(0-12)	OS	6.05[1.52-24.07]	P	7
Zhang 2016[27]	HK2	China	Asian	NPC	52.49(3.75-93.63)	140	107/33	IHC	I-IV	Score>3(0-12)	OS DMFS	1.72[0.46-6.38] 1.26[0.36-4.45]	P	7
											RFS	3.71[0.04-349.71]		
Zhou 2017[28]	GLUT1	China	Asian	NPC	36	63	41/22	IHC	I-IV	>2	OS	1.72[0.88-3.34]	P	7
Zhu 2014[29]	MCT4	China	Asian	OSCC	NA	99	59/40	IHC	I-IV	Score≥6(0-7)	OS DFS	3.64[1.60-8.29] 3.42[1.51-7.78]	P	7
Zuo 2016[30]	GLUT1	China	Asian	LSCC	NA	57	47/10	IHC	I-IV	NA	OS	1.97[0.12-33.19]	P	6

M/F: male/female; cut-off value: value that distinguishes high and low expression of glycolysis markers; HR: hazard ratio; CI: confidence interval; TMA: tissue microarray; IHC: Immunohistochemistry; P: prospective; NA: not available; OSCC: oral squamous cell carcinoma; LSCC: laryngeal squamous cell carcinoma; SCC: squamous cell carcinoma; cSCC: cutaneous squamous cell carcinoma; IF: Immunofluorescence staining; NPC: nasopharyngeal carcinoma; TTSCC: squamous cell carcinoma of the tonsil and mobile tongue.

### Glycolysis markers and OS in HNSCC

**Figure 2:**
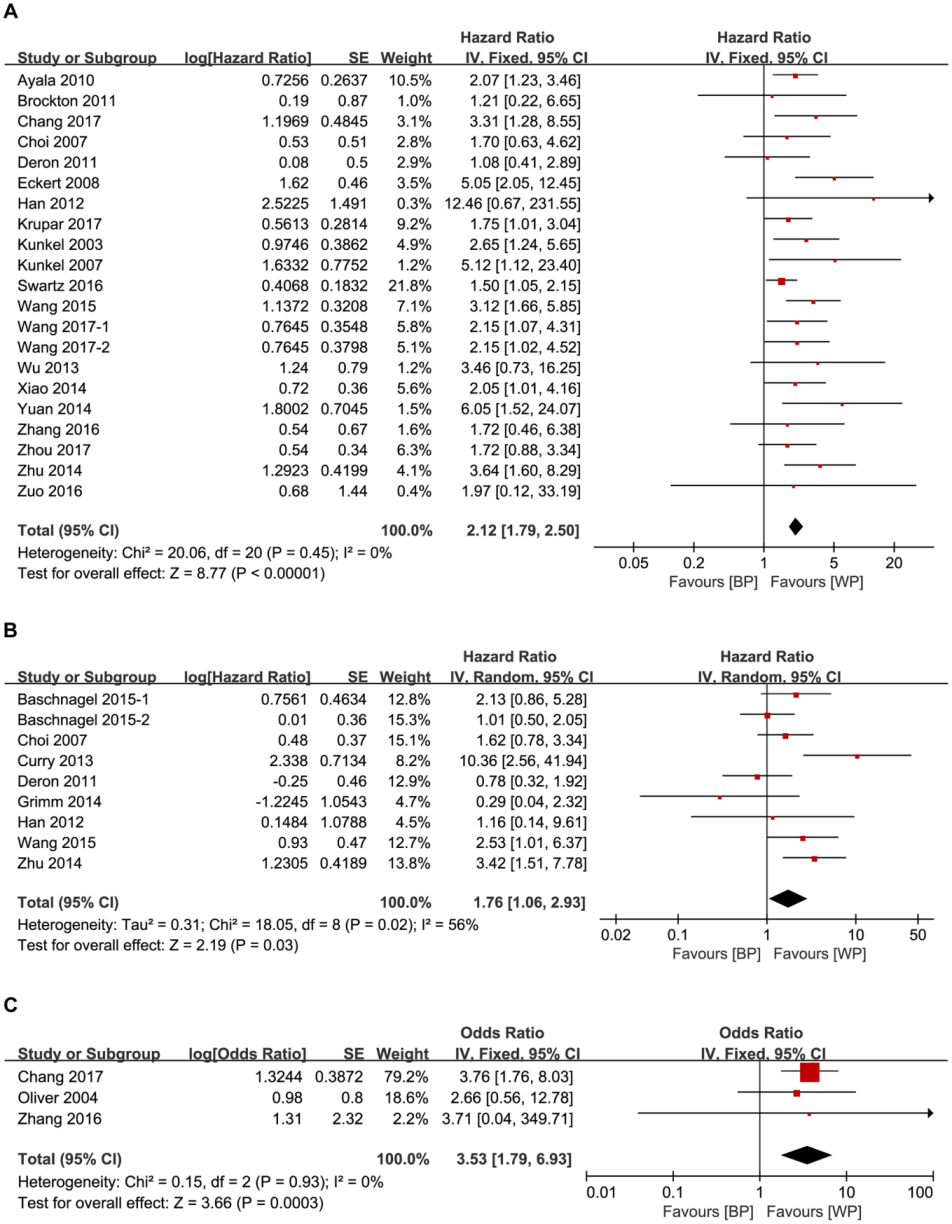
Forest plots show the association between the expression levels of glycolysis markers and (**A**) OS (**B**) DFS and (**C**) RFS of HNSCC patients. Note: BP: better prognosis; WP: worse prognosis.

We evaluated the relationship between expression levels of the 5 glycolysis markers and OS in HNSCC using data from twenty-one studies that included 1893 patients [14–34]. High expression of glycolysis markers correlated with poor OS of HNSCC patients (HR = 2.12, 95% CI: 1.79-2.50, *P* < 0.001; Figure 2A). There was no significant heterogeneity between the studies (I^2^ = 0%, *P*_h_ =0.45; Figure 2A).

### Glycolysis markers and DFS in HNSCC

We analyzed the relationship between the 5 glycolysis markers and DFS in HNSCC using data from nine studies that included 771 patients [17, 21, 29, 31, 35–38]. High expression of glycolysis markers significantly correlated with poor DFS (HR = 1.76, 95% CI: 1.06-2.93, *P* = 0.03), but there was significant heterogeneity between the analyzed studies (I^2^ = 56%, *P*_h_ = 0.02; Figure 2B) [18].

### Glycolysis markers and RFS in HNSCC

We then analyzed the data from three studies with 284 patients [16, 27, 39] to determine the relationship between RFS and the expression levels of HK2, GLUT1 and GLUT4 in HNSCC tissues. We observed significant correlation between expression levels of the three glycolysis markers and RFS (HR = 3.53, 95% CI: 1.79-6.93, *P* < 0.001). Moreover, there was no significant heterogeneity between the three studies (I^2^ = 0%, *P*_h_ = 0.93; Figure 2C).

### Glycolysis markers and DSS or DMFS in HNSCC

We analyzed the data from two studies with 132 patients [20, 40] to determine the relationship between DSS and the expression levels of GLUT1 and MCT4 in HNSCC patients. We also analyzed data from two studies with 198 patients [27, 41] to determine the relationship between DMFS and the expression levels of GLUT1 and HK2 in HNSCC patients. We did not observe any significant correlation between DFS and the expression levels of GLUT1 and MCT4. Moreover, the relationship between DMFS and the expression levels of GLUT1 and HK2 was inconclusive because the data varied significantly between the two studies (Table 1).

### Subgroup analysis

To explore the potential source of heterogeneity, we performed subgroup analyses by stratifying data based on ethnicity, glycolysis markers, tumor location, and sample sizes (Table 2).

**Table 2:** Prognostic value of glycolysis markers in HNSCC patients.

	**Variable**	**Study NO.**	**Sample size**	**HR [95% CI]**	***P* value**	**Heterogeneity**	
						**I**^2^ **(%)**	***P* value**
OS	Overall	21	1893	2.12 [1.79, 2.50]	<0.001	0	0.45
	**Ethnicity**						
	Asian	12	1039	2.53 [1.93, 3.30]	<0.001	0	0.80
	Caucasian	9	854	1.89 [1.52, 2.34]	<0.001	22	0.24
	**Glycolysis marker**						
	GLUT1	12	998	1.94 [1.57, 2.39]	<0.001	2	0.42
	MCT4	1	99	3.64 [1.60, 8.29]	0.002	-	-
	HK2	3	324	2.05 [1.29, 3.26]	0.002	0	0.96
	PKM2	3	311	2.92 [1.85, 4.59]	<0.001	0	0.41
	GLUT4	2	161	1.93 [0.97, 3.81]	0.06	61	0.11
	**Tumor location**						
	OSCC	11	982	2.71 [2.12, 3.46]	<0.001	0	0.60
	NPC	3	250	1.85 [1.17, 2.91]	0.008	0	0.93
	LSCC	2	106	3.04 [0.78, 11.80]	0.11	0	0.73
	Oropharyngeal SCC	1	274	1.50 [1.05, 2.15]	0.026	-	-
	**Sample size**						
	Large	10	1321	2.06 [1.69, 2.50]	<0.001	0	0.50
	Small	11	566	2.29 [1.66, 3.17]	<0.001	13	0.32
DFS	Overall	9	771	1.76 [1.06, 2.93]	0.03	56	0.02
	**Ethnicity**						
	Asian	4	303	2.24 [1.42, 3.54]	<0.001	0	0.52
	Caucasian	5	468	1.46 [0.61, 3.49]	0.39	70	0.01
	**Glycolysis marker**						
	GLUT1	4	351	1.55 [0.92, 2.64]	0.10	1	0.39
	MCT4	2	141	4.55 [2.24, 9.23]	<0.001	44	0.18
	HK2	1	97	1.01 [0.50, 2.05]	0.98	-	-
	PKM2	1	111	2.53 [1.01, 6.37]	0.008	-	-
	GLUT4	1	71	0.78 [0.32, 1.92]	0.59	-	-
	**Tumor location**						
	OSCC	6	506	2.33 [1.18, 4.60]	0.01	52	0.06
	**Sample size**						
	Large	5	565	1.75 [0.94, 3.27]	0.08	53	0.07
	Small	4	206	1.88 [0.68, 5.18]	0.22	68	0.02

Subgroup analysis based on ethnicity showed that high expression levels of the five glycolysis markers correlated with poorer OS in Asians (HR = 2.53, 95% CI: 1.93-3.30, *P* < 0.001) and Caucasians (HR = 1.89, 95% CI: 1.52-2.34, *P* < 0.001). However, higher expression levels of glycolysis markers were associated with poorer DFS only in Asians (HR = 2.24, 95% CI: 1.42-3.54, *P* < 0.001).

We further stratified data based on the expression levels of individual glycolysis markers and found that higher expression levels of GLUT1 (HR = 1.94, 95% CI: 1.57-2.39, *P* < 0.001), MCT4 (HR = 3.64, 95% CI: 1.60-8.29, *P* = 0.002), HK2 (HR = 2.05, 95% CI: 1.29-3.26, *P* = 0.002), and PKM2 (HR = 2.92, 95% CI: 1.85-4.59, *P* < 0.001) correlated with poorer OS in HNSCC patients. Moreover, higher expression of MCT4 (HR = 4.55, 95% CI: 2.24-9.23, *P* < 0.001), and PKM2 (HR = 2.53, 95% CI: 1.01-6.37, *P* = 0.008) correlated with worse DFS in HNSCC patients.

We then performed subgroup analysis based on the location of HNSCC and found that higher expression levels of the five glycolysis markers were associated with poorer OS in patients with oral squamous cell carcinoma (OSCC) (HR = 2.71, 95% CI: 2.12-3.46, *P* < 0.001), nasopharyngeal carcinoma (NPC) (HR = 1.85, 95% CI: 1.17-2.91, *P* = 0.008), and oropharyngeal SCC (HR = 1.50, 95% CI: 1.05-2.15, *P* = 0.026). Moreover, OSCC patients with higher expression of glycolysis markers were associated with worse DFS (HR = 2.33, 95% CI: 1.18-4.60, *P* = 0.01).

Furthermore, HNSCC patients with higher expression levels of glycolysis markers in both large sample size (HR = 2.06, 95% CI: 1.69-2.50, *P* < 0.001) and small sample size (HR = 2.29, 95% CI: 1.66-3.17, *P* < 0.001) groups were associated with worse OS.

**Figure 3:**
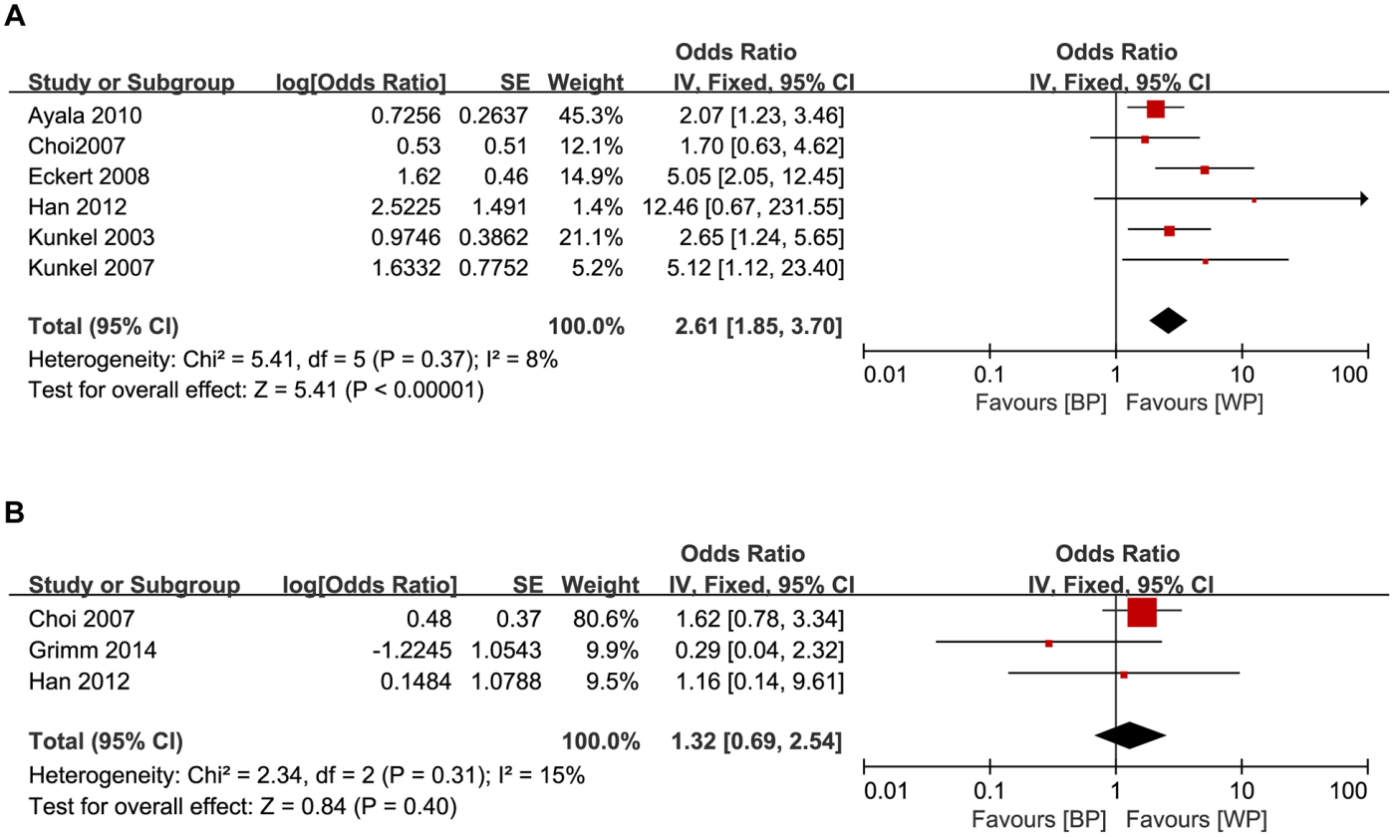
Forest plots show the relationship between the levels of GLUT1 expression and (**A**) OS and (**B**) DFS in OSCC patients. Note: BP: better prognosis; WP: worse prognosis.

We also analyzed the relationship between GLUT1 expression levels and OS or DFS in OSCC patients. OSCC patients with higher GLUT1 levels were associated with poorer OS (HR = 2.61, 95% CI: 1.85-3.70, *P* < 0.001; Figure 3A), but did not show significant association with DFS (HR = 1.32, 95% CI: 0.69-2.54, *P* = 0.40; Figure 3B).

### Galbraith plot analysis

**Figure 4:**
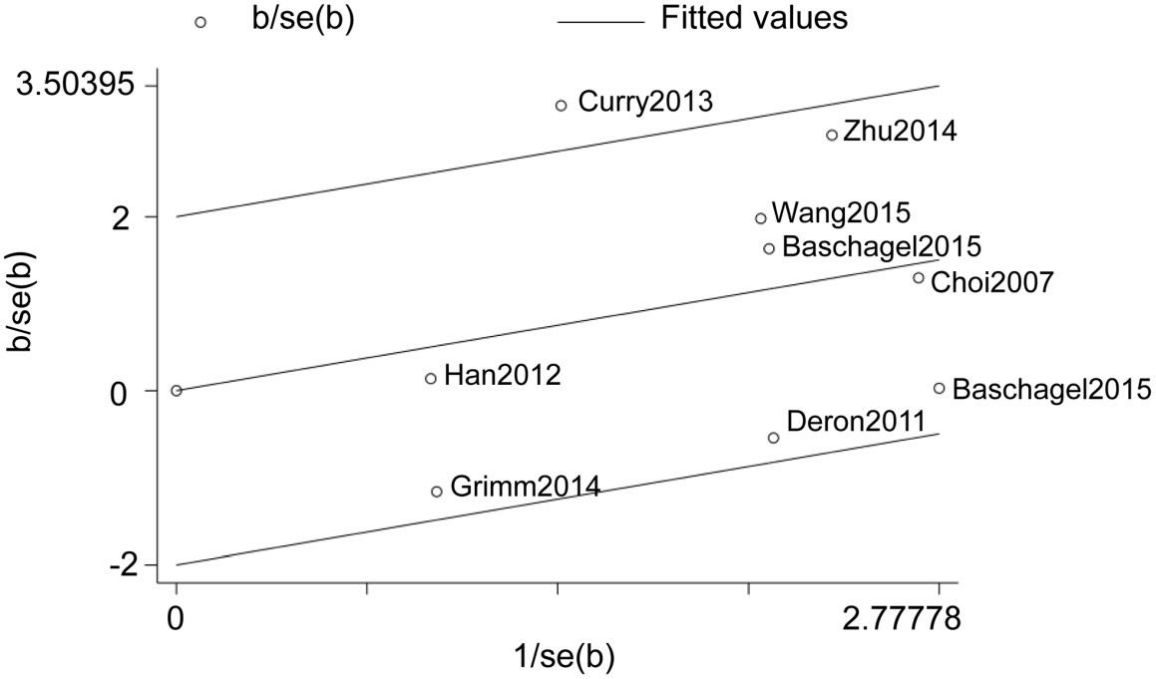
**Galbraith plot analysis shows the source of heterogeneity in studies regarding the association between the expression levels of glycolysis markers and DFS in HNSCC patients.**

**Figure 5:**
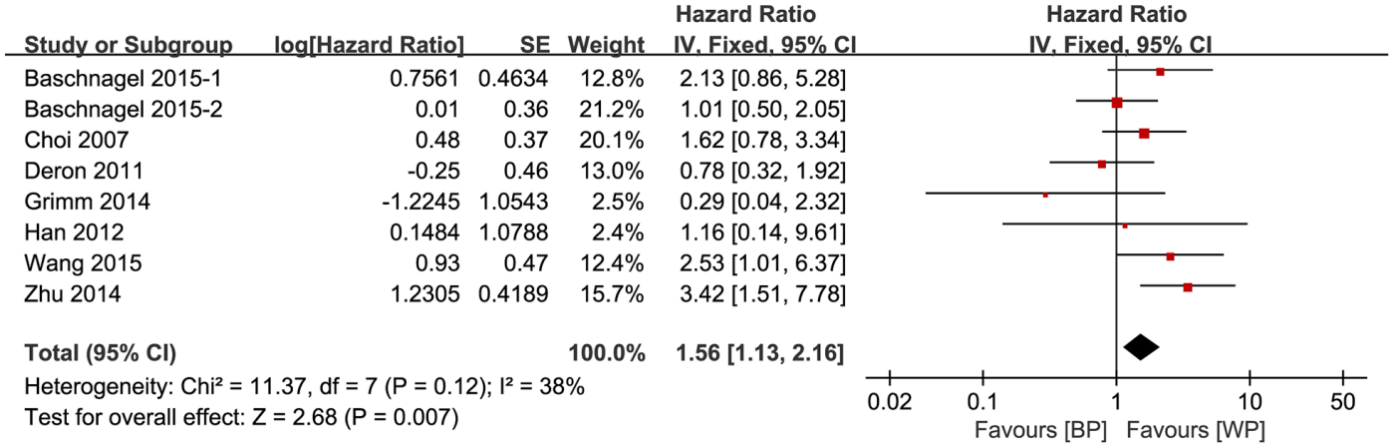
**Association between the expression levels of glycolysis markers and DFS after removing the source of heterogeneity.**

We then constructed Galbraith plots to investigate the source of heterogeneity in studies describing the expression levels of glycolysis markers and DFS. Results showed that there was one study by Curry et al. [36] outside the CI and thus might be the source of heterogeneity (Figure 4). There was no statistically significant heterogeneity detected (I^2^ = 38%, *P*_h_ = 0.12) after removing this study, but the significant relationship between glycolysis markers’ expression and DFS was not changed (HR = 1.56, 95% CI: 1.13-2.16, *P* = 0.007; Figure 5).

### Sensitivity analysis

We performed sensitivity analyses to further investigate the relationship between the expression levels of glycolysis markers and the OS and DFS of HNSCC patients based on fifteen [14, 16, 18, 21–24, 26–29, 31, 32, 34, 38] and seven [21, 29, 31, 35, 37, 38] high-quality studies (NOS score ≥ 7, Table 3), respectively. In most cases, the data revealed similar trends as reported in the subgroup analyses. However, the sensitivity analysis showed that the expression levels of glycolysis markers did not correlate with OS in NPC patients (HR = 1.72, 95% CI: 0.95-3.11, *P* = 0.07) or DFS in HNSCC patients (HR = 1.55, 95% CI: 0.97-2.47, *P* = 0.07).

**Table 3:** Sensitivity analysis of high-quality studies to determine the prognostic value of glycolysis markers in HNSCC patients.

	**Variable**	**Study NO.**	**Sample size**	**HR [95% CI]**	***P* value**	**Heterogeneity**	
						**I**^2^ **(%)**	***P* value**
OS	Overall	15	1596	2.14 [1.78, 2.57]	<0.001	15	0.29
	**Ethnicity**						
	Asian	10	949	2.50 [1.91, 3.27]	<0.001	0	0.76
	Caucasian	5	647	2.04 [1.35, 3.08]	<0.001	52	0.08
	**Glycolysis marker**						
	GLUT1	7	748	1.92 [1.52, 2.43]	<0.001	22	0.26
	MCT4	1	99	3.64 [1.60, 8.29]	0.002	-	-
	HK2	2	277	2.04 [1.11, 3.78]	0.02	0	0.77
	PKM2	3	311	2.92 [1.85, 4.59]	<0.001	0	0.41
	GLUT4	2	161	1.93 [0.97, 3.81]	0.06	61	0.11
	**Tumor location**						
	OSCC	9	909	2.69 [2.07, 3.48]	<0.001	0	0.52
	NPC	2	203	1.72 [0.95, 3.11]	0.07	0	1
	LSCC	1	49	3.46 [0.73, 16.25]	0.12	-	-
	Oropharyngeal SCC	1	274	1.50 [1.05, 2.15]	0.026	-	-
	**Sample size**						
	Large	9	1248	2.10 [1.71, 2.59]	<0.001	0	0.44
	Small	6	348	2.26 [1.53, 3.36]	<0.001	41	0.13
DFS	Overall	7	696	1.55 [0.97, 2.47]	0.07	47	0.08
	**Ethnicity**						
	Asian	3	270	2.32 [1.45, 3.70]	<0.001	0	0.40
	Caucasian	4	426	1.08 [0.68, 1.71]	0.76	29	0.24
	**Glycolysis marker**						
	GLUT1	3	318	1.59 [0.92, 2.74]	0.10	33	0.23
	MCT4	1	99	3.42 [1.51, 7.78]	0.003	-	-
	HK2	1	97	1.01 [0.50, 2.05]	0.98	-	-
	PKM2	1	111	2.53 [1.01, 6.37]	0.008	-	-
	GLUT4	1	71	0.78 [0.32, 1.92]	0.59	-	-
	**Tumor location**						
	OSCC	4	431	2.09 [1.33, 3.31]	0.002	45	0.14
	**Sample size**						
	Large	5	565	1.75 [0.94, 3.27]	0.08	53	0.07
	Small	2	131	1.21 [0.69, 2.13]	0.50	35	0.22

### Publication bias

**Figure 6:**
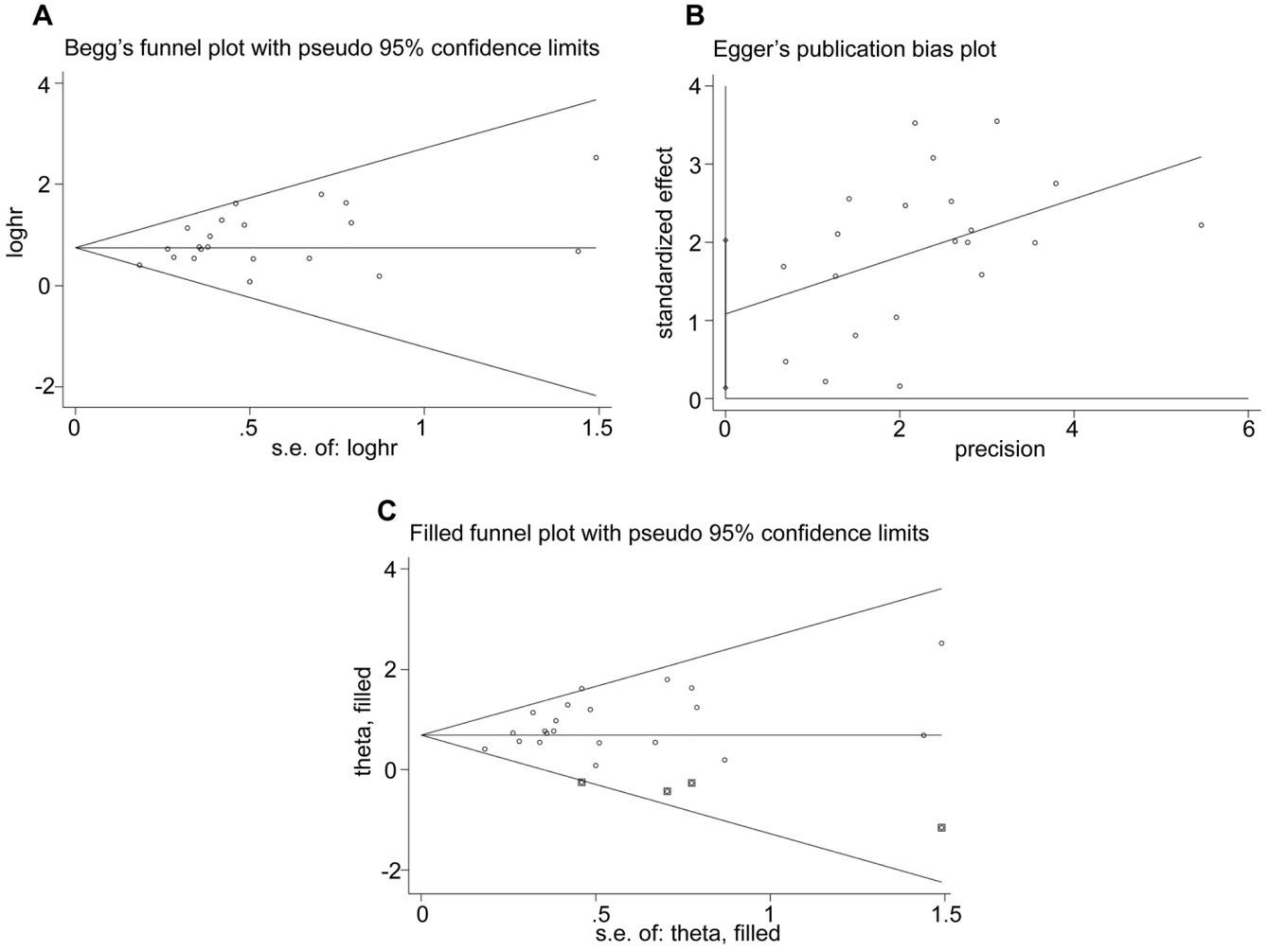
**Evaluation of publication bias.** (**A**) Begg’s funnel plots, (**B**) Egger’s test and (**C**) Funnel plots adjusted using trim and fill method show the evaluation of publication bias among studies used to assess the relationship between the expression levels of glycolysis markers and OS.

We then analyzed publication bias in studies regarding OS using Begg’s funnel plots and Egger’s test. Begg’s test did not show significant publication bias among the included studies (*P* = 0.156, Figure 6A). However, Egger’s test showed significant publication bias (*P* = 0.027, Figure 6B). Furthermore, the funnel plots were adjusted by using trim and fill method. The results were not significantly altered after adding 4 suppositional studies (HR = 0.683, 95% CI: 0.521–0.846; Figure 6C), indicating that our result was robust.

## DISCUSSION

Cancer cells generate significant amounts of energy through aerobic glycolysis to sustain rapid proliferation, migration, survival, and chemotherapeutic drug resistance [42, 43]. Several signaling pathways such as AKT/mTOR, AMP-activated protein kinase (AMPK), NF-κB, and HIF-1α regulate glycolysis [11, 42, 44]. Glycolysis is upregulated in many types of cancers, but systematic meta-analysis of the relationship between the expression of glycolysis markers and prognosis of HNSCC patients has not been carried out.

In this meta-analysis, we analyzed data from 37 studies with 3272 patients to determine the prognostic value of five glycolysis markers in HNSCC. Our results showed that high expression levels of glycolysis markers in HNSCC tissues significantly correlated with worse OS, DFS, and RFS of HNSCC patients. These results are consistent with the results of most studies included in this meta-analysis.

Stratified analysis by ethnicity revealed that higher expression of glycolysis markers was associated with shorter OS in both Asians and Caucasians. However, their higher expression correlated with poorer DFS only in Asians. These results suggest that the roles of these glycolysis markers in HNSCC growth and progression may vary among different ethnic populations. The results of our meta-analysis may help clinicians stratify HNSCC patients into appropriate high- and low-risk categories based on the expression of glycolysis markers.

Sensitivity analysis by sample size suggested that higher expression of glycolysis markers predicted unfavorable OS, but was not associated with DFS in both small and large sample size HNSCC patient groups. The heterogeneity between studies in the DFS analysis suggested the need for more clinical studies to overcome bias. Furthermore, higher expression of these glycolysis markers was linked to worse OS and DFS in OSCC and poorer OS in oropharyngeal SCC, but was not associated with OS or DFS in NPC or laryngeal squamous cell carcinoma (LSCC). This suggested that the requirement of glycolysis markers may vary in different types of HNSCC. Moreover, our results suggest that these glycolysis markers may be potential therapeutic targets in OSCC and oropharyngeal SCC patients.

The members of the glucose transporter (GLUT) family are upregulated in several cancer types and mediate glucose uptake that is required to sustain the high energy demand required by cancer cells for various biochemical programs. Our results showed that higher expression of GLUT1 predicted poorer OS in HNSCC patients. This was consistent with previous findings in other solid tumors [45]. A study by Chang et al. reported that upregulation of GLUT4 in HNSCC patients was associated with poorer overall survival and recurrence-free survival [16]. However, our meta-analysis did not demonstrate association between high GLUT4 expression and OS or DFS in HNSCC patients. This may be attributed to the smaller sample size in the studies included in our meta-analysis.

Hexokinase 2 (HK2) catalyzes phosphorylation of glucose into glucose-6-phosphate and represents the first rate-limiting step of glycolysis [11]. Previous studies showed that high expression of HK2 was significantly correlated with poorer OS in various solid tumors [46]. Consistent with these reports, we found that higher HK2 expression was significantly linked to shorter OS in HNSCC patients.

MCT4 plays a vital role in monocarboxylic acid export [10] and PKM2 catalyzes the last step of glycolysis by converting phosphoenolpyruvate into pyruvate with the generation of a molecule of ATP [47]. Our meta-analysis showed that higher MCT4 and PKM2 expression was associated with poorer OS and DFS in HNSCC patients. However, sample sizes in the analyses of MCT4 and PKM2 were relatively small. Therefore, further large sample size studies are required to confirm our findings. Previous studies have shown that higher MCT1 and PKM2 expression levels are associated with poor prognosis in several cancers [10, 42].

Overall, these results suggest that GLUT1, HK2, PKM2 and MCT4 are potential therapeutic targets to improve survival outcomes of HNSCC patients.

The results of our meta-analysis showed that higher GLUT1 expression correlated with worse OS in OSCC patients. These results are consistent with the results from a previous study [48]. Higher GLUT1 expression strongly correlated with a more invasive, proliferative, and malignant OSCC, which is associated with poorer prognosis [49–51].

This meta-analysis has several limitations. Firstly, we identified significant heterogeneity among studies related to DFS, but these effects could not be eliminated or explained completely. Galbraith plot demonstrated that the study by Curry et al. [36] contributed significantly towards heterogeneity in studies regarding DFS. The heterogeneity was eliminated after removing this study from the analysis. However, we observed heterogeneity in sensitivity analysis that may have been caused by differences in the age and tumor stages of patients in different studies that were used for this meta-analysis. Secondly, half of the studies were carried out in Asian patients and the remaining patients were of Caucasian origin. This may have resulted in population selection bias. Thirdly, the included studies were all prospective and may have contributed to bias. Lastly, we observed publication bias in our meta-analysis. We adjusted funnel plots using trim and fill method and the results were not significantly changed after adding 4 suppositional studies, thereby demonstrating the robustness of our analysis. However, additional large-scale, high-quality, long-term studies are necessary to confirm the findings of our meta-analysis.

In conclusion, our meta-analysis demonstrates that glycolytic pathway enzymes are potential prognostic biomarkers and therapeutic targets in HNSCC patients. Overall, high expression of the four glycolysis markers in the tumor tissues correlated with poorer OS, DFS, and RFS in HNSCC patients.

## MATERIALS AND METHODS

### Literature selection and inclusion criteria

We performed literature search in the PubMed, Web of Science and Cochrane Library databases between January 2000 and August 2020 without restrictions on the type of publications or the study regions for the following MeSH headings in the title or abstract: (monocarboxylate transporter 4 OR hexokinase 2 OR glucose transporter 1 OR glucose transporter 4 OR pyruvate kinase M2) AND ((head and neck squamous cell carcinoma OR HNSCC OR ((oral OR larynx OR pharynx OR tongue OR oropharynx OR nasopharynx OR hypopharynx OR trachea OR laryngopharynx OR cervical tracheal OR cervical esophagus) AND (cancer OR tumor OR carcinoma OR neoplasm))).

### Inclusion and exclusion criteria

Available studies were included according to the following criteria: 1) the relationship between glycolysis marker expression and overall survival (OS), disease-free survival (DFS), recurrence-free survival (RFS), disease-specific survival (DSS) or distant metastasis-free survival (DMFS) in HNSCC was described; 2) HRs and 95% CIs could be obtained or estimated from the data provided in the text; 3) the diagnosis of HNSCC was done according to pathological examination.

The exclusion criteria were: 1) editorials, reviews, case reports, letters to the editor; 2) animal experimental studies; 3) sample size < 30 samples.

### Data extraction

Two authors (YW and YL) independently extracted data regarding author, publication time, study country and ethnicity, tumor location, follow-up period, sample size, TNM stage, cut-off values of glycolysis markers, and survival data from the included studies. The HRs and 95% CIs were either reported in the included studies or estimated from raw data using Kaplan-Meier survival curve analysis [52, 53]. Any disagreement between the two authors was resolved by a senior author (JX).

### Study quality assessment

The quality of all included studies was assessed by the Newcastle-Ottawa Scale (NOS) [54]. They were allocated a score of 0-9 independently by two authors (YW and YL). Any disagreements between the two authors were resolved by discussion. The studies with a score of seven or above were considered as of high quality.

### Statistical analysis

The meta-analysis was performed according to the Cochrane Collaboration and the Quality of Reporting of Meta-analyses guidelines [55, 56]. The hazard ratio (HR) was considered as a summary statistic for censored outcomes (OS, DFS, RFS, DSS, DMFS) [52]. HNSCC patients with HR values greater than 1 were considered to be associated with poor survival.

Heterogeneity between studies was evaluated using chi-square test and a *P* value less than 0.10 was considered as significant heterogeneity. I^2^ statistic was used to quantify heterogeneity. A random-effects model was used to evaluate studies with heterogeneity, whereas those without heterogeneity were evaluated using the fixed-effects model [57]. Galbraith plot analysis was performed to identify the studies with heterogeneity.

We used the median value of all samples as the boundary between the large and small sample size. Subgroup analyses were performed based on different glycolysis markers, ethnicity, sample sizes, and tumor locations. Sensitivity analysis was conducted for high quality studies. Publication bias was evaluated using Begg’s test and Egger’s test and the credibility of the results was evaluated using the trim and fill method [58]. The statistical analyses were performed using Review Manager version 5.3 (Cochrane Collaboration, Copenhagen, Denmark) and STATA SE version 12.0 (Stata Corporation, College Station, TX, USA). A two-tailed *P* < 0.05 was considered statistically significant.

## Supplementary Material

Supplementary Table 1
