# Supplementary material for: Prognostic value of glycolysis markers in head and neck squamous cell carcinoma: a meta-analysis
Source: Aging (Albany NY). 2021 Feb 26;13(5):7284–99. doi: 10.18632/aging.202583 (PMC7993722; doi:10.18632/aging.202583)
Supplement: Supplementary Table 1 [file aging-13-202583-s001.pdf]

SUPPLEMENTARY TABLE

Supplementary Table 1. Risk of bias in the prospective studies using modified Newcastle-Ottawa Scale.

Study	Selection				Comparability	Outcome			Quality score
	Representativeness of the exposed cohort	Selection of the non-exposed cohort	Ascertainment of exposure	Demonstration that outcome of interest was not present at start of study	Comparability of cohorts on the basis of the design or analysis	Assessment of outcome	Long enough follow-up for outcomes to occur	Adequacy of follow-up of cohorts	
Ayala 2010[14]	0	1	1	1	2	0	1	1	7
Baschnagel 2015[35]	0	1	1	1	2	0	1	1	7
Brockton 2011[15]	0	1	0	1	2	0	1	1	6
Chang 2017[16]	0	1	1	1	2	1	1	1	8
Choi 2007[31]	0	1	1	1	2	0	1	1	7
Curry 2013[36]	0	1	1	1	2	0	1	1	7
Deron 2011[38]	0	1	1	1	2	0	1	1	7
Eckert 2008[32]	0	1	1	1	2	0	1	1	7
Eckert 2011[40]	0	1	1	1	2	0	1	1	7
Grimm 2014[37]	0	1	1	1	2	1	1	1	8
Han 2012[17]	0	1	1	1	0	0	1	1	5
Jonathan 2006[41]	0	1	1	1	2	1	1	1	8
Krupar 2017[19]	0	1	0	1	2	0	1	1	6
Kunkel 2003[33]	0	1	1	1	2	0	1	1	7
Kunkel 2007[34]	0	1	0	1	2	0	1	1	6
Oliver 2004[39]	0	1	1	1	1	0	1	1	6
Swartz 2016[18]	0	1	1	1	2	0	1	1	7
Sweeny 2012[20]	0	1	1	1	2	0	1	1	7
Wang 2015[21]	0	1	1	1	2	0	1	1	7
Wang 2017-1[22]	0	1	1	1	2	0	1	1	7
Wang 2017-2[23]	0	1	1	1	2	0	1	1	7
Wu 2013[24]	0	1	1	1	2	0	1	1	7
Xiao 2014[25]	0	1	0	1	0	0	1	1	4
Yuan 2014[26]	0	1	1	1	2	0	1	1	7
Zhang 2016[27]	0	1	1	1	2	0	1	1	7
Zhou 2017[28]	0	1	1	1	2	0	1	1	7
Zhu 2014[29]	0	1	1	1	2	0	1	1	7
Zuo 2016[30]	0	1	0	1	2	0	1	1	6
